# Microsporidian infection of mosquito larvae changes the host-associated microbiome towards the synthesis of antimicrobial factors

**DOI:** 10.1186/s13071-025-06813-z

**Published:** 2025-05-17

**Authors:** Artur Trzebny, Abigail D. Taylor, Jeremy K. Herren, Johanna K. Björkroth, Sylwia Jedut, Miroslawa Dabert

**Affiliations:** 1 Molecular Biology Techniques Laboratory, Faculty of Biology, Adam Mickiewicz University, Poznan, Poland; 2 International Centre of Insect Physiology and Ecology, Nairobi, Kenya; 3 Department of Food Hygiene and Environmental Health, Faculty of Veterinary Medicine, University of Helsinki, Helsinki, Finland

**Keywords:** Microsporidia, Parasites, Microbiota, Mosquitoes, Host–microbe interactions, Disease vectors, Biosynthetic pathways, Antimicrobial factors, *Wolbachia pipientis*, *Weissella viridescens*

## Abstract

**Background:**

Microsporidians (Microsporidia) are a group of obligate intracellular parasites that commonly infect mosquitoes. Recently, it has been shown that infection by these parasites can alter the composition and functionality of the mosquito-associated microbiome. The host-associated microbiome of the mosquito can play a pivotal role in various physiological processes of this host, including its vector competence for pathogens. Thus, understanding how microsporidians shape the mosquito microbiome may be crucial for elucidating interactions between these parasites and their mosquito hosts, which are also vectors for other parasites and pathogens.

**Methods:**

The effects of microsporidian infection on the microbiome structure and functionality of *Culex pipiens* and *Culex torrentium* larvae under semi-natural conditions were examined. The host-associated microbiome of *Cx*. *pipiens* (*n* = 498) and *Cx*. *torrentium* (*n* = 465) larvae, including that of the 97 infected individuals of these samples, was analysed using 16S DNA profiling and functional prediction analysis.

**Results:**

Microbiome network analysis revealed that, in the microsporidian-positive larvae, host microbial communities consistently grouped within a common bacterial module that included Aerococcaceae, Lactobacillaceae, Microbacteriaceae, Myxococcaceae, and Polyangiaceae. Indicator species analysis revealed two strong positive correlations between microsporidian infection and the presence of *Weissella* cf. *viridescens* and *Wolbachia pipientis*. Functional predictions of microbiome content showed enrichment in biosynthetic pathways for ansamycin and vancomycin antibiotic groups in infected larvae. Furthermore, the MexJK-OprM multidrug-resistance module was exclusively present in the infected larvae, while carbapenem- and vancomycin-resistance modules were specific to the microsporidian-free larvae.

**Conclusions:**

Our results demonstrate that microsporidian infection alters the microbial community composition in mosquito larvae. Moreover, they show that microsporidian infection can increase the antimicrobial capabilities of the host-associated microbiome. These results provide novel insights into host microbiome-parasite interactions and have potential implications for the vector competencies of mosquitoes.

**Graphical abstract:**

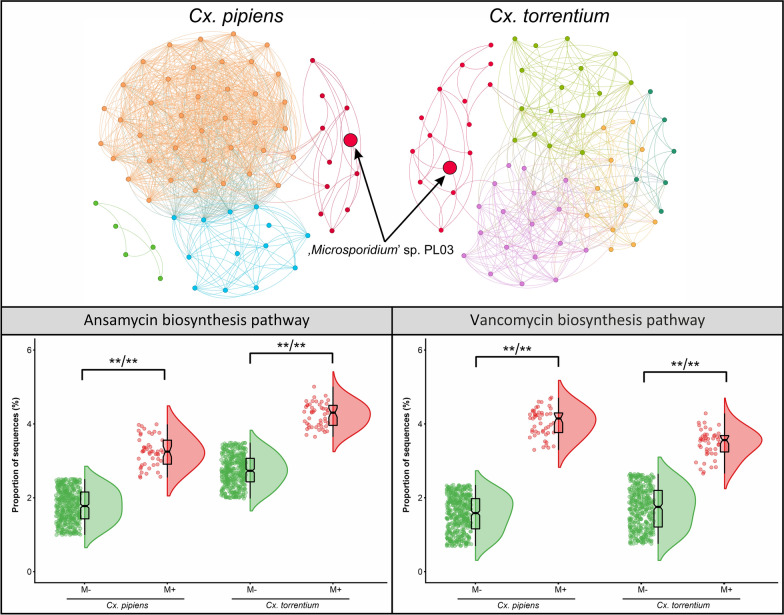

**Supplementary Information:**

The online version contains supplementary material available at 10.1186/s13071-025-06813-z.

## Background

The host-associated microbiome plays a central role in several physiological processes of the host, including vector competence for parasites and pathogens. The microbiome has been shown to mediate the fermentation of non-digestible substrates to produce short-chain fatty acids, regulate glucose and energy homeostasis, metabolise bile acids as signalling molecules, maintain aerobic balance in the gut, and produce specific metabolites, such as trimethylamine and indolepropionic acid [1]. As a dynamic and complex ecosystem, the microbiome is integral to maintaining host homeostasis, influencing processes such as digestion, pathogen defence, and immune system regulation [2–5]. In addition to providing metabolic functions, the microbiome contributes to the synthesis of bioactive compounds, including amino acids, vitamins, and short-chain fatty acids, which are critical for host health, development, and survival [6–9].

Mosquitoes (Culicidae) are haematophagous arthropods and vectors of numerous human and animal pathogens, and their microbiome plays pivotal and multifaceted roles. It mediates blood meal digestion, modulates the immune response, and exerts a significant influence on pathogen transmission dynamics [10–14]. For example, the bacteria *Serratia* spp. and *Chromobacterium* sp. Csp_P strain, and the yeast *Wickerhamomyces anomalus*, which are naturally associated with mosquitoes, produce lytic enzymes and toxin metabolites that directly impact *Plasmodium* spp., reducing their capacity to develop in *Anopheles* and *Aedes* mosquitoes [15–18].

Similarly, it has been shown that the endosymbiotic bacteria of the genus *Wolbachia*, which is ubiquitously distributed in mosquito populations, may impair mosquito competence for virus transmission via pathogen interference in their invertebrate hosts [19–22]. This effect may be mediated by competition for key metabolic resources within mosquito cells, such as cholesterol and amino acids [23–27]. Additionally, *Wolbachia* infections trigger critical immune signalling pathways, including Toll-like receptor signalling, the immune deficiency pathway, and Janus kinase/signal transducer and activator of transcription cascades, which enhance mosquito immune defences against pathogens [27–31].

Mosquitoes can be infected by microsporidians (Microsporidia), which are obligate intracellular eukaryotic parasites [32]. Among the more than 1500 described species, over 250 species, representing 34 genera, have been reported in mosquitoes, including human-pathogenic *Encephalitozoon hellem* [33–35]. Microsporidians primarily colonize the adipose tissue and epithelium of the digestive system, though they have been known to infect a range of tissues, such as muscle, germline, and excretory system tissues [34]. Although microsporidians are considered typical parasites, some mutualistic interactions have been proposed for these organisms, such as an influence on host fitness through faster larval development, higher adult emergence, and larger body size [36–38]. Additionally, some studies have reported that the microsporidian species *Microsporidia* MB, which was recently discovered in Africa, impairs the development of *Plasmodium falciparum* in *Anopheles arabiensis* [39].

Even though microsporidians have been the subject of extensive research for decades, reports on their ability to modulate the host-associated microbiota have only appeared recently [40–48]. In honeybees (*Apis cerana* and *Apis* *mellifera*), infection by *Vairimorpha ceranae* significantly decreased the abundances of fungi belonging to the genera *Podosphaera* and *Blumeria*, and bacteria belonging to the genus *Bifidobacterium* and the family Pasteurellaceae, whereas it increased the abundances of the bacteria belonging to the genera *Rosenbergiella* and *Serratia* [41–43]. In wild bumblebees (*Bombus terrestris*), a bacterium of the genus *Snodgrassella* was associated with *Nosema bombi* infections [44, 45]. In silkworms (*Bombyx mori*), infection by *Nosema bombycis* resulted in a significant decline in microbiota diversity, while *Enterocytozoon hepatopenaei* infection in shrimps resulted in a proliferation of pathogenic bacteria [47–49].

However, in mosquitoes, the effects of microsporidians on the host microbiome remain largely understudied [40]. It has been shown that microsporidian infection in field-collected adult mosquitoes correlates with changes in the composition and function of the microbiome. The infected mosquitoes exhibited the exclusive presence of the bacteria *Weissella* cf. *viridescens* and a strong association with *Spiroplasma* sp. [50]. Moreover, the infection of *Aedes aegypti* larvae by *Edhazardia aedis* resulted in a significant increase in the *Serratia marcescens* bacterial load [51]. Recently, functional predictions for the microbiome in microsporidian-infected adult mosquitoes indicated an enrichment of metabolic pathways related to defence functions and synthesis of precursors of nucleotides, suggesting that microsporidians may influence host metabolism through microbiome modulation [50].

In adult mosquitoes, the microbiome is shaped mainly by diet and migration. However, in the case of mosquito larvae, the composition of the microbiome is predominantly influenced by the aquatic environment where they live and find food [13, 52–55]. This environmental dependency makes the larval model especially valuable for studying interactions among parasites and the host-associated microbiome. Thus, the aim of this study was to investigate the impact of microsporidian infection on the mosquito larvae microbiome, focusing on changes in the structure and functionality of the microbiome. Using semi-natural experimental conditions to minimise external biotic and abiotic factors, we sought to elucidate the dynamics of host-associated microbiome modulation during a microsporidian infection [52, 53].

## Methods

### Materials

For this study, DNA isolates from a total of 963 *Culex pipiens* (*n* = 498) and *Culex torrentium* (*n* = 465) larvae, collected for a previous study [56] between July and August 2022 from Morasko, Poznan, Poland (52°28′03.7"N 16°55′59.8"E), were used. The larvae had been collected from a 50-L barrel by using a larval dipper and individually preserved in 96% ethanol immediately after collection, without being reared or maintained as a colony in the laboratory. Forty-six of the 498 (9.2%) *Cx. pipiens* and 47 of the 465 (10.1%) *Cx. torrentium* larvae were positive for one microsporidian species,'*Microsporidium*'sp. PL03. A DNA sample extracted from 20 L of the water in which the larvae had been living, and a blank DNA extraction, were used as negative controls and were analysed together with the tested samples.

### Microbial community profiling

For microbial community profiling, we used the V4 region of the 16S ribosomal RNA (rRNA) gene. Detailed information on the polymerase chain reaction (PCR) protocol and amplicon sequencing is described in a previous study [50]. Briefly, for the PCR, we used the 515 F (GTGCCAGCMGCCGCGGTAA) and 806R (GGACTACHVGGGTWTCTAAT) primers [57] tailed at the 5′-ends with dual-indexed Ion Torrent A and P adapters. Sequencing was performed using the Ion 540 Kit-OT2 and Ion 540 chip on the Ion S5 system (Life Technologies, USA), according to the manufacturer’s protocol, with a planned minimum 100,000 reads per sample analysed.

### Read processing and data analysis

Short reads (< 180 base pairs) were removed from the dataset using Geneious Prime version 2023.1.2 (Biomatters). We used the FastX-Toolkit [58] to extract sequences with > 50% of bases having a quality score ≥ 25. Sequences were divided by indexes in Geneious Prime and trimmed at the 5′- and 3′-ends to remove PCR primers. Amplicon sequencing variants (ASVs) were generated using the DADA2 denoise-pyro method implemented in QIIME2 version 2024.10 [59, 60]. ASVs detected in control samples were removed from the dataset using the UNCROSS2 algorithm [61]. Thereafter, ASVs were compared with reference sequences in the SILVA database using ARB for small subunit ribosomal RNAs version 138.1 [www.arb-silva.de] [62–64].

The functional potential of the microbial communities was predicted by the Phylogenetic Investigation of Communities by Reconstruction of Unobserved States (PICRUSt2) package version 2.4.1 [65]. The ASV abundances were normalised using information about 16S rRNA gene copy numbers in each taxon. The resulting ASV table was used for metagenome functional prediction by generating a table of Kyoto Encyclopedia of Genes and Genomes (KEGG) orthologs [66–68]. The predictions were categorised according to KEGG orthology levels 1, 2, and 3 within the hierarchical structure of the KEGG pathway database. To assess the accuracy of the PICRUSt2 predictions, the nearest sequenced taxon index was estimated and calculated for each sample [69].

### Identification of *Weissella* species

*Weissella* species were confirmed by PCR amplification and Sanger sequencing of a 689-base pair fragment of the 16S rRNA gene using S-G-Wei-0121-a-S-20 and S-G-Wei-0823-a-A-18 primers [70], as described previously [50].

### Statistical analyses

The Shannon diversity index for individual samples was calculated using the vegan package version 2.6–8 [71] and compared using a* t*-test for independent means as well as one-way ANOVA with post hoc Tukey’s honestly significant difference test. A Bray–Curtis-based principal coordinates analysis was employed to analyse the microbial community compositions. Additionally, ANOVA was used to detect differences in the microbiome composition between infected and non-infected larvae. For determining multivariable association between microbiomes, linear models implemented in MaAsLin2 were used [72]. Indicator species analysis [73] was conducted to evaluate whether microsporidian species occurred exclusively during specific seasons, and whether their presence was consistently associated with particular treatment groups, as determined by the A and B components of the analysis. The indicator species analysis, based on 9 × 10^10^ permutations, was performed using the multipatt function in the indicspecies package version 1.7.15 [73, 74]. Pearson’s correlation coefficient (*r*) [75] was calculated to determine the correlations between detected taxa. The ecological networks were calculated based on Spearman’s correlation matrix using the psych package version 2.4.6.26 [76]. To reduce the complexity of the network and enhance the resolution of core microbiome community detection, only significant (*P* < 0.05) taxa were visualised using the Fruchterman–Reingold layout with an area value of 10,000 and gravity value of 10 in Gephi version 0.10.1 [77].

## Results

### Host-associated microbiota of* Culex* larvae

In total, 107 bacterial families (18 phyla) were identified from the analysed samples including the water sample, which represented the larval aquatic habitat (Table S1). Of these 107 families, 24 were exclusive to mosquito larvae, 37 were exclusive to the aquatic habitat, and 44 were common to *Culex* spp. larvae and their aquatic habitat (Fig. S1). Corynebacteriaceae and Micrococcaceae were exclusive to *Cx. pipiens*, while 37 families were specific for the water sample.

**Fig. 1:**
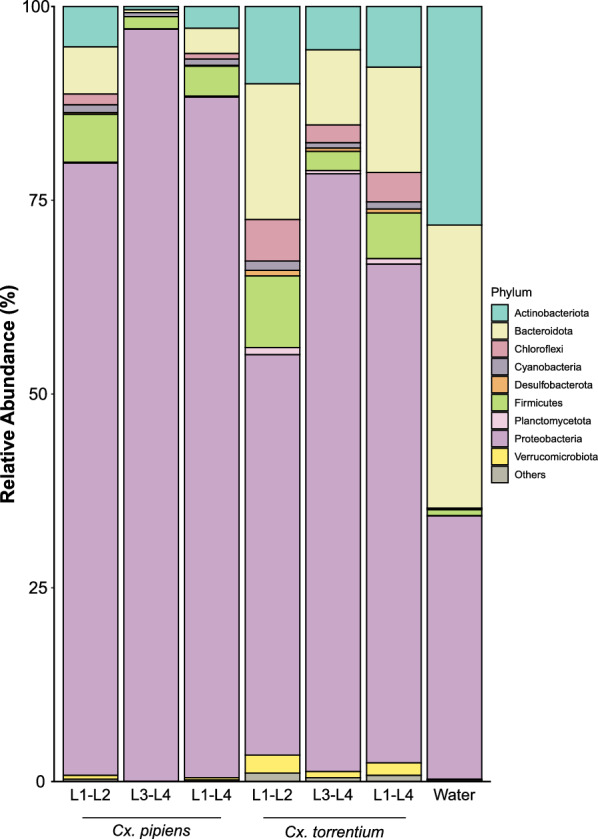
Boxplot showing the relative abundances of the phyla identified in the examined samples

Although general differences in the Shannon diversity between infected and non-infected hosts were not significant (*T* = 1.18; *P* = 0.8), a slight but statistically significant difference was observed between *Cx. pipiens* samples (Tukey’s honestly significant difference = 4.13; *P* = 0.02) (Fig. S2). Proteobacteria (*Cx. pipiens*, 88.33%; *Cx. torrentium*, 72.9%), Bacteroidota (*Cx. pipiens*, 2.49%; *Cx. torrentium*, 11.49%), and Firmicutes (*Cx. pipiens*, 2.54%; *Cx. torrentium*, 3.88%) were the most abundant phyla associated with both mosquito species. In the water sample, which represented the larval habitat, Bacteroidota (35.23%), Proteobacteria (34.11%), and Actinobacteriota (28.54%) were the most abundant phyla (Fig. 1; Table S2).

**Fig. 2:**
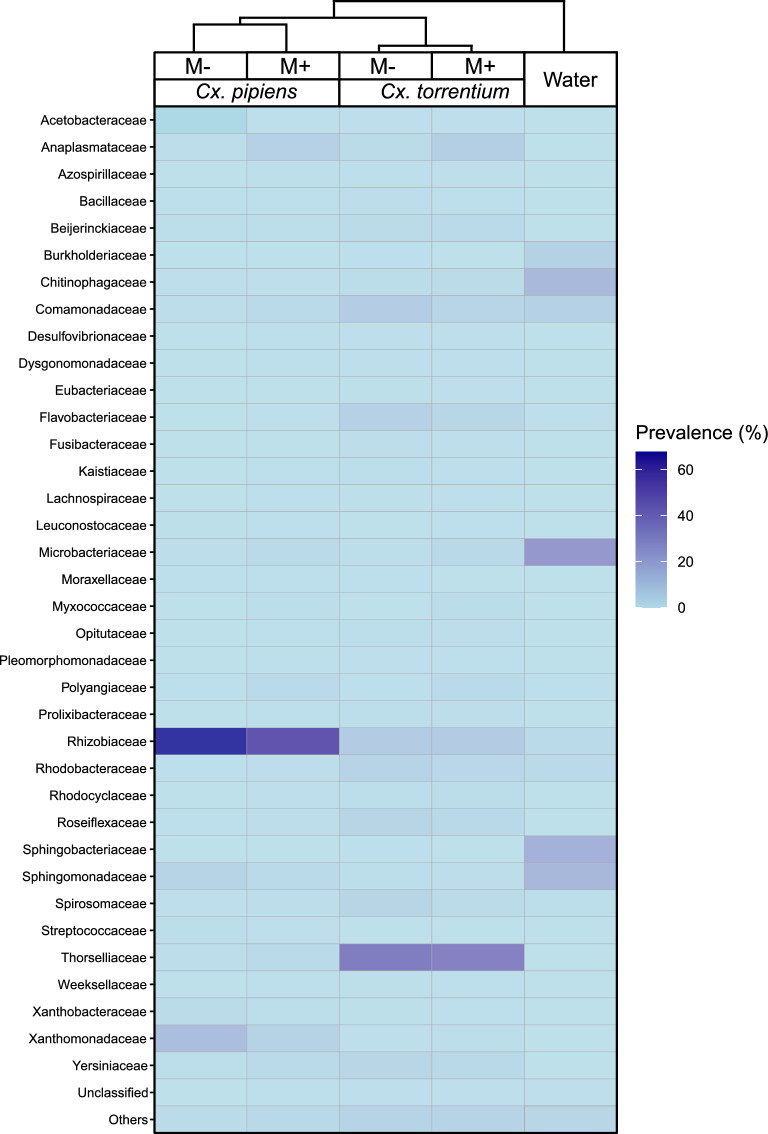
Hierarchical clustering heatmap showing the prevalence (%) of bacterial families in* Culex pipiens* and* Culex torrentium* samples positive (*M*+) or negative (*M*−) for microsporidians, and in water samples. Colour intensity corresponds to prevalence, with* light blue* indicating low prevalence and* dark blue* indicating high prevalence

The microbiota of non-infected *Cx. pipiens* was dominated by Rhizobiaceae (67.73%), Xanthomonadaceae (12.84%), and Sphingomonadaceae (4.68%), while that of infected *Cx. pipiens* individuals was dominated by the families Rhizobiaceae (56.65%), Anaplasmataceae (5.36%), and Xanthomonadaceae (5.04%). Bacteria belonging to Thorselliaceae (39.69%), Rhizobiaceae (7.63%), and Comamonadaceae (6.96%) formed the main core of the microbiota of non-infected *Cx. torrentium* larvae (Fig. 2; Table S1). Thorselliaceae and Rhizobiaceae were also the most abundant families in the microsporidian-positive *Cx. torrentium*, reaching a similar level of 37.91% and 7.53% sequence reads, respectively. The third most dominant family in infected *Cx. torrentium* individuals was the Anaplasmataceae, which reached a level of 6.71%. Cluster analysis using the unweighted pair group method with arithmetic mean demonstrated that the diversity of bacterial communities was driven by mosquito species rather than by microsporidian infection (Fig. 2).

There were no significant differences in the microbiota composition between infected and non-infected larvae of either mosquito species (*F* = 0.001; *P* = 0.977). However, specific bacterial taxa displayed significant differences (*P* < 0.01) in relative abundance between these groups (Table S3). In *Cx. pipiens*, families such as Xanthomonadaceae, Corynebacteriaceae, and Xanthobacteraceae were more characteristic for non-infected larvae (coefficients = 1.35, 1.26, and 1.01, respectively). Conversely, the families Lachnospiraceae, Pleomorphomonadaceae, Azospirillaceae, and Myxococcaceae were significantly less characteristic for non-infected larvae (negative coefficients = 3.58, 3.91, 4.17, and 5.24, respectively). In *Cx. torrentium*, non-infected larvae were enriched with Enterococcaceae (coefficient = 2.0), Bacillaceae (coefficient = 1.86), Bryobacteraceae (coefficient = 1.66), and Sphingobacteriaceae (coefficient = 1.58). Polyangiaceae (coefficient = 3.08), Leuconostocaceae (coefficient = 3.27) and Myxococcaceae (coefficient = 4.91) were significantly less abundant.

**Fig. 3:**
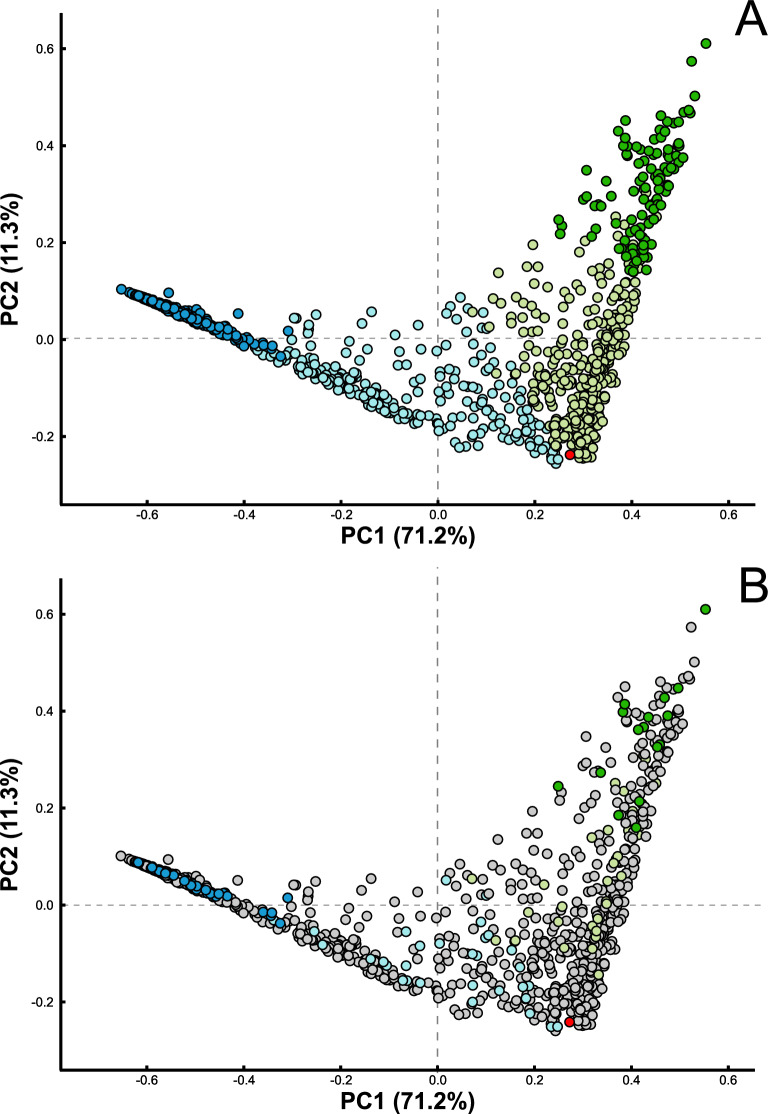
Principal coordinate (*PC*) analysis of Bray–Curtis dissimilarity based on microbial community composition. **A** Samples grouped by microbiota composition from early (*light green*) and late (*dark green*) larval stages of* Culex pipiens*, early (*light blue*) and late (*dark blue*) larval stages of* Culex torrentium*, and water (*red*). **B** Samples colour coded by ‘*Microsporidium*’ sp. PL03 infection status, where microsporidian-positive individuals (*M*+) are shown in* green* (*Cx. pipiens*),* blue* (*Cx. torrentium*), and* red* (*water*), while non-infected individuals (*M*−) are shown in* grey*

Principal component analysis also confirmed that the larval microbiota was strongly determined by mosquito species (Fig. 3A). Early larval stages of *Cx. pipiens* and *Cx. torrentium* exhibited microbiota resembling those of the water habitat, but as development progressed, host-associated microbiota became more species specific. Overall, microsporidian infection did not significantly affect the overall microbiota structure, as microbiota of infected larvae did not cluster distinctly (Fig. 3B).

### Relationship between host-associated microbiota composition and microsporidian infection

**Fig. 4:**
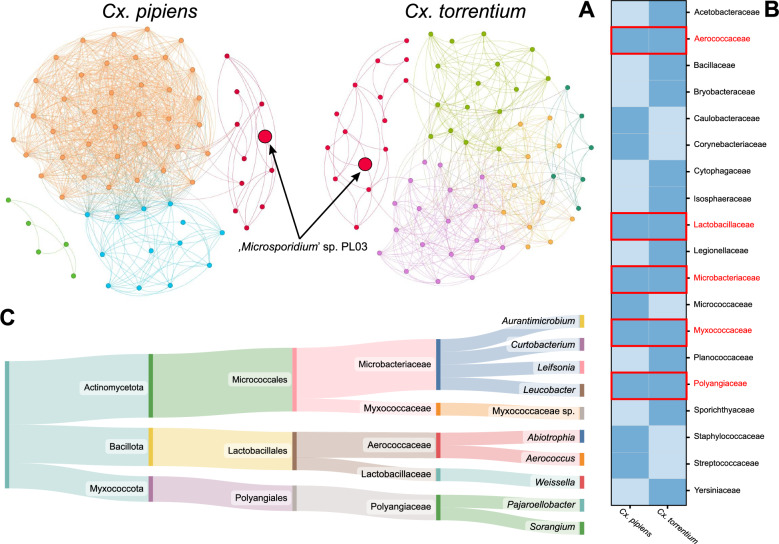
**A** Co-occurrence networks of microbial communities in* Culex pipiens* and* Culex torrentium*, from ‘*Microsporidium*’ sp. PL03-positive individuals. Nodes represent bacterial families, with significant co-occurrence relationships (*P* < 0.05) are shown as edges. **B** Presence or absence of bacterial families is shown within the red module for both* Cx. pipiens* and* Cx. torrentium*. Colour intensity indicates prevalence, with* dark blue* denoting presence and* light blue* denoting absence of a given bacterial family. Taxa identified in both species are marked with a* red frame*. **C** Sankey diagram showing the systematics of the common taxa in the module containing'*Microsporidium*'sp. PL03

Network analysis of the microbiome composition of microsporidian-positive *Cx. pipiens* and *Cx. torrentium* larvae revealed notable differences in bacterial community complexity and composition. The microbiome network of *Cx. pipiens* exhibited a highly complex architecture (edges = 885; average degree = 26.029) characterised by a dense network of interactions (density = 0.388) and four major modules (Fig. 4A). In contrast, the network of *Cx. torrentium* displayed reduced complexity (edges = 391; density = 0.182), with fewer connections per node (average degree = 11.848) and five distinguishable modules (Fig. 4A). In both *Culex* species, microsporidian was consistently associated with a shared (red) module (Fig. 4A).

Across clusters within the module containing microsporidian, 19 bacterial families were identified, five of which (Aerococcaceae, Lactobacillaceae, Microbacteriaceae, Polyangiaceae, and Myxococcaceae) were common to both mosquito species (red boxes in Fig. 4B). Among these, 10 species were detected: *Abiotrophia* sp., *Aerococcus* sp. (Aerococcaceae), *W.* cf. *viridescens* (Lactobacillaceae), *Aurantimicrobium* sp., *Curtobacterium* sp., *Leifsonia* sp., *Leucobacter* sp. (Microbacteriaceae), *Pajaroellobacter* sp., *Sorangium* sp. (Polyangiaceae), and one species belonging to the Myxococcaceae (Fig. 4C).

**Fig. 5:**
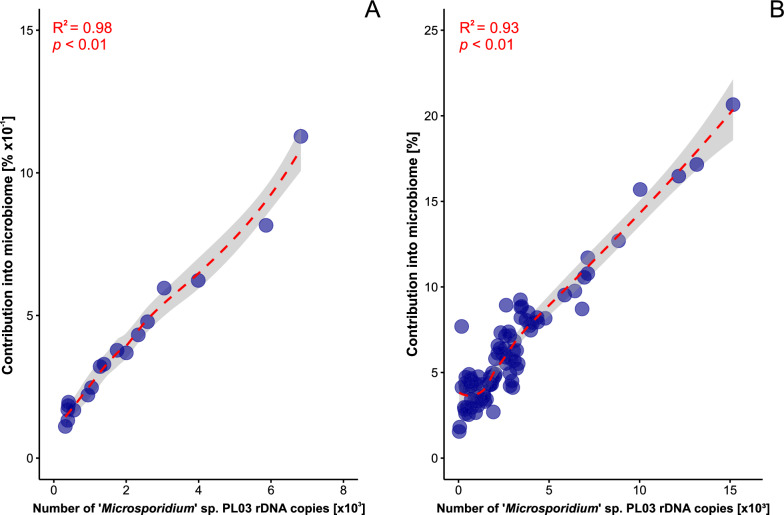
Relationship between the number of'*Microsporidium*'sp. PL03 ribosomal DNA copies and **A** contribution of* Weissella* cf.* viridescens* and **B*** Wolbachia* between the* Culex* spp.* Dashed red lines* represent fitted regression models, with* shaded areas* indicating the 95% confidence intervals

Indicator species analysis for microsporidian-positive mosquitoes showed that *W.* cf. *viridescens* displayed high values for the exclusive taxon component (*A* = 1 and *B* = 0.18; *P* = 0.05), and thus a significantly strong positive correlation was observed (*R* = 0.988; *P* < 0.001) (Fig. 5A; Table S4). The remaining taxa components of'*Microsporidium*'sp. PL03 module showed slight negative or positive correlations, but these relationships were not statistically significant (Table S4). Beyond this module, *Wolbachia*, an exclusive taxon from the Anaplasmataceae, displayed a strong significant positive correlation with microsporidian infection (*R* = 0.95; *P* < 0.001) (Fig. 5B; Table S4).

### Relationship between host-associated microbiome function and microsporidian infection

Using the 16S amplicon dataset, PICRUSt2 analysis identified 156 functional pathways based on KEGG pathway metadata. The predicted metagenomes exhibited nearest sequenced taxon index scores ranging from 0.0001 to 0.293, with mean values of 0.062 and 0.058 for infected and non-infected mosquitoes, respectively.

Among the KEGG orthology level 2 pathways, carbohydrate metabolism, amino acid metabolism, and the metabolism of cofactors and vitamins were the most prevalent in both infected and non-infected *Culex* species. Each of these pathways exceeded 10% in relative abundance (Fig. S3; Table S5). Other metabolic pathways, related to metabolism of lipids, terpenoids, and polyketides, constituted 6–8% each, while less abundant pathways, including energy metabolism, glycan biosynthesis, and xenobiotics biodegradation, ranged from 3 to 6% each. The other pathways contributed < 3% to the predicted metagenomic profiles each (Fig. S3; Table S5). Hierarchical clustering revealed that microbial functional profiles were primarily determined by mosquito species, with infection status exerting only a minor effect on certain pathways, such as xenobiotics biodegradation and lipid metabolism (Fig. S3).

**Fig. 6:**
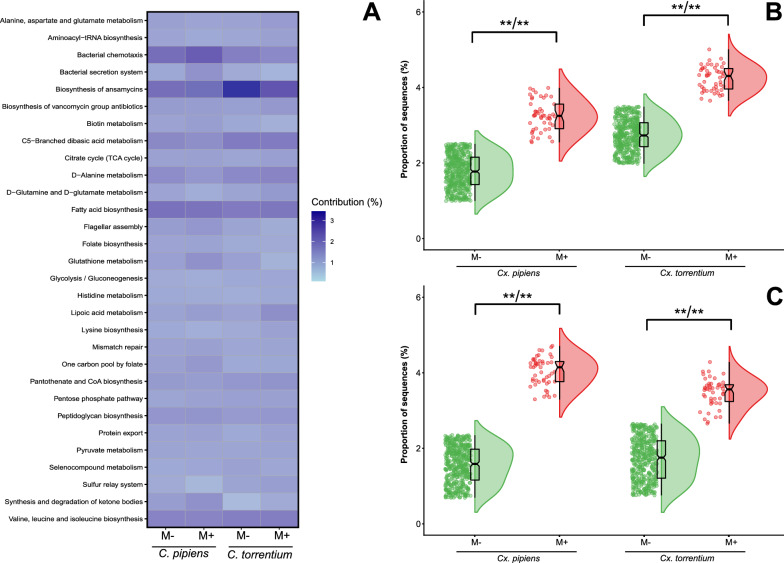
**A** Hierarchical clustering heatmap showing the prevalence (%) of the most prevalent functional metabolic profiles at level 3 of the microbiome in* Culex pipiens* and* Culex torrentium* infected by ‘*Microsporidium*’ sp. PL03 (*M*+) and non-infected ones (*M*−). Colour intensity reflects prevalence, with* light blue* indicating low prevalence and* dark blue* indicating high prevalence. Raincloud plots for the proportion of sequences assigned to the activity of the ansamycin (**B**) and vancomycin biosynthesis pathways (**C**) in* Cx. pipiens* and* Cx. torrentium* infected by ‘*Microsporidium*’ sp. PL03 (*M*+) and non-infected ones (*M*-)

A total of 155 biological pathways at level 3 were reconstructed in both *Culex* species, of which 154 belonged to *Cx. pipiens* and 152 to *Cx. torrentium* (Fig. S4; Table S6). Among the 30 most relevant pathways, approximately 80% (76.67%) were associated with metabolism and 13.3% with genetic information processing. The remaining 10% were associated with cellular processes and environmental information processing (6.67% and 3.33%, respectively) (Fig. 6A; Table S7). Among the dominant pathways, significant changes were observed in four pathways of microsporidian-positive larvae of both *Culex* species. Ansamycin biosynthesis activity was elevated nearly twofold in infected *Cx. pipiens* and 1.5-fold in infected *Cx. torrentium*, while vancomycin biosynthesis increased by 2.6-fold and twofold, respectively (Fig. 6B; Table S7). The difference in the pentose phosphate pathway between infected and non-infected individuals was < 0.05% and was not statistically significant. Glutathione metabolism decreased by 1.2-fold in infected *Cx. pipiens* but increased by 1.4-fold in infected *Cx. torrentium*. Conversely, aminoacyl transfer RNA biosynthesis increased by over onefold in *Cx. pipiens* and decreased by a similar magnitude in *Cx. torrentium* (Table S7).

Thirteen signature modules (i.e. functional units of gene sets) associated with drug resistance were identified through the reconstruction of signalling modules in the *Culex* larval microbiome (Table S8). Among them, 10 were present in both infected and non-infected mosquitoes. A common signature module unique to microsporidian-positive individuals of both species was multidrug-resistance efflux pump MexJK-OprM. Non-infected mosquitoes were characterised by modules linked to carbapenem and vancomycin resistance, including the D-Ala-D-Lac type module (Fig. S5).

## Discussion

Our results show that the core microbial composition of mosquito larvae consists of Proteobacteria (approximately 80%), Bacteroidota (approximately 7%) and Firmicutes (approximately 5%). This distribution reflects the dominant phyla frequently observed across mosquito microbiota and aligns with previous studies, where Actinobacteria, Bacteroidota, Proteobacteria, and Firmicutes were reported as the most abundant phyla in *Aedes aegypti* and *Mansonia* spp. larvae. Together they make up approximately 90% of the microbiota [78–80]. Similarly, Proteobacteria, Cyanobacteria, Bacteroidetes, and Firmicutes were the most abundant phyla in *Culex tarsalis* [54].

Our study revealed that early-instar larvae of *Culex* spp. shared bacterial community structure with that of their surrounding aquatic environment. However, as their development progressed, the composition of their host-associated microbiota became dissimilar, and was specific to the mosquito species. This observation supports the hypothesis that the mosquito larval microbiota closely mirrors microbial communities in the water habitat during early developmental stages, but diverges as larvae age [54, 78, 81]. This is confirmed by the greater diversity of microbial taxa in early-stage larvae, which suggests that microbial filtering and selection processes occur during larval development, reducing diversity by the late-instar stages [54, 78, 82]. *Culex pipiens* exhibited higher network connectivity in their microbiota composition, whereas *Cx. torrentium* displayed lower microbial connectivity. Notably, these observations for *Cx. pipiens* align with findings from laboratory studies of *Culex pipiens* f. *molestus* and *Culex quinquefasciatus*, where the microbiota network of *Cx. pipiens* f. *molestus* similarly demonstrated greater complexity and stronger correlations than that of *Cx. quinquefasciatus* [83]. These differences may be influenced by host-specific factors, environmental factors, or distinct ecological interactions, such as those related to resource availability or competition.

We found positive correlations between microsporidian infection and specific host-associate microbiota, such as *Weissella* cf. *viridescens* and *Wolbachia*, which further highlight the intricate interplay between host microbiota and microsporidians. The occurrence of *W*. cf. *viridescens* in *Culex* larval microbiota was unequivocally associated with microsporidian infection. This is consistent with results of a previous study [50] conducted on adult mosquitoes collected in the field, where *W*. cf. *viridescens* was detected exclusively in microsporidian-positive individuals. One reason for this is that infection-induced acidification of the intestinal lumen environment creates conditions favourable for colonization by *Weissella*, which is a lactic acid bacterium well adapted to low-pH environments [84, 85]. This hypothesis aligns with the results of studies demonstrating that microsporidian infections reduce gut pH, creating a selective environment for acid-tolerant species such as *Weissella* spp. [84, 85]. However, the dynamics of *Weissella* spp. abundance under pathogenic stress remain complex. For example, studies on interactions between *Weissella confusa* (Bacilli: Lactobacillaceae) and *Paranosema locustae* (Microsporidia) in migratory locust (*Locusta migratoria*) suggest a transient increase in *W. confusa* abundance during early infection stages, followed by a decline as pathogenic stress intensifies. These findings indicate a dual role for *Weissella*: a transient opportunist or a long-term protective symbiont. Moreover, reductions in *Weissella* abundance have been correlated with increased host mortality, suggesting potential protective effects mediated by competitive exclusion, antimicrobial metabolite production such as that of bacteriocins, and stabilisation of the gut microbiome under stress [86]. Further research that measures gut pH and explores the functional contributions of *Weissella* spp. in infected hosts is warranted to elucidate its role in the interplay between host, pathogen, and the host-associated microbiome. Such findings could expand our understanding of the ecological roles of lactic acid bacteria in gut ecosystems under pathogenic stress and their potential application in managing insect health in the context of biological control strategies.

The presence of microsporidians was also strongly positively related to the prevalence of *Wolbachia*. The observed positive correlation between the abundance of microsporidia and *Wolbachia* may be explained by an indirect mechanism in which oxidative stress plays a pivotal role. Infection with microsporidia, such as *Vairimorpha ceranae* or *Encephalitozoon cuniculi*, induces the production of reactive oxygen species, leading to oxidative stress in host cells. In response, the host activates antioxidant enzymes, including catalase and glutathione S-transferase, to neutralise reactive oxygen species and protect cellular structures [87–90]. Previous studies have demonstrated that elevated levels of antioxidants promote the proliferation of *Wolbachia*, which benefit from the enhanced redox environment of the host [31]. Oxidative stress induced by microsporidian infection might exhibit tissue specificity, as evidenced by the high proliferation of *Wolbachia* in the gut. However, further investigations focusing on immune responses, particularly Toll-like receptor signalling and immune deficiency pathways, leading to oxidative stress responses [91], as well as *Wolbachia* proliferation in different tissues under microsporidian infection, are warranted to further elucidate these mechanisms.

Our metagenomic analyses suggest that microsporidian infection alters the functional potential of the mosquito microbiome, increasing the abundance of bacterial taxa associated with the synthesis of the antibiotics ansamycin (approximately 1.8-fold) and vancomycin (approximately 2.3-fold). These results align with those of a previous study conducted on field-collected adult mosquitoes, which exhibited an approximately 1.6-fold upregulation in the ansamycin biosynthetic pathway and a 1.8-fold upregulation in the vancomycin biosynthetic pathway [50]. In contrast to the present study, the activity of the pentose phosphate pathway showed no differences between infected and non-infected larvae; although a slight overall increase in pentose phosphate pathway activity was observed in the microsporidian-positive larvae analysed, the difference between infected and non-infected individuals was negligible. This suggests that the mosquito microbiome adopts a defensive strategy by producing antibiotics, such as ansamycins and vancomycin. Therefore, by producing these antibiotics, the microbiome probably limits opportunistic pathogen invasion and enhances host resilience [92].

A heightened capacity to produce antibiotics appears to be a natural defence mechanism that helps to protect the host, which has been weakened by parasitic infection. Ansamycins are a group of bacterial macrocyclic polyketides known for their wide range of inhibitory effects, including antibacterial, antifungal, antiviral, and immunosuppressive properties [93–95]. In contrast, vancomycin is a glycopeptide antibiotic with a branched tricyclic structure that disrupts cell wall synthesis of Gram-positive bacteria [96–98]. The increased production of antibiotics, particularly ansamycins, may also reflect a competitive strategy within the gut niche, where microsporidia impose selective pressures on the microbial community by competing for limited resources and space. In response, bacteria synthesize secondary metabolites, such as ansamycins, not only to inhibit or eliminate competitors but also to modulate the gut microenvironment in a manner that enhances their survival and ecological fitness [99–102].

Additionally, reconstruction of multidrug-resistance efflux pump MexJK-OprM in the *Culex* larval microbiome suggested that microsporidian infections induce oxidative stress in mosquitoes, disrupting cellular homeostasis, including quorum sensing signalling within the microbiome [87, 90]. This disturbance may activate bacterial efflux pump (MexJK) activity, which is known to modulate siderophore production and iron homeostasis [103–105]. Activation of MexJK may facilitate the efflux of siderophores or other iron-regulatory molecules, increasing the availability of free iron in the mosquito host, and thus, indirectly benefit intracellular microsporidia, which require iron for replication and proliferation [90]. However, it is important to note that this proposed mechanism remains hypothetical and warrants further investigation. Targeted approaches, for instance, such as genetic manipulation of bacterial efflux pumps and host-microbe co-culture models under controlled oxidative conditions, can be utilised for the experimental validation of this hypothesis.

Overall, our study highlights the complex and dynamic interactions within the host-associated microbiome. Future research should focus on disentangling the mechanistic underpinnings of these interactions, particularly the functional roles of *Weissella* cf. *viridescens* and *Wolbachia*, to better understand their potential applications in vector and pathogen control. These insights may inform the development of targeted microbiome-based strategies for managing mosquito-borne diseases.

## Conclusions

We demonstrated that microsporidian infection alters the composition of microbial communities in mosquito larvae. We showed that, during microsporidian infection, host microbial communities consistently grouped with microsporidians within a common bacterial module that included the same five bacterial families. Additionally, *Weissella viridescens* and *Wolbachia pipientis* were strongly positively correlated with the presence of microsporidians. This result corroborates earlier observations that microsporidians modulate the gut microbiome in mosquitoes, and underscores their significance in host microbial networks and host microbiome-parasite interactions. Moreover, the results suggest that microsporidian infection can enhance antimicrobial capabilities by increasing the synthesis of the antibiotics ansamycin and vancomycin in infected mosquito larvae. This finding may be pertinent to the prospect of using microsporidians as biological agents to restrict the development of *Plasmodium* or *Dirofilaria* in mosquito tissues. However, further research is warranted to substantiate this hypothesis. This research should also determine actual metabolic activity, and experimental infections under controlled conditions should be conducted to test the hypothesis.

## Supplementary Information

Additional file 1

## Data Availability

Data generated in this study are available from GenBank under accession nos. OR500623-OR500624 and PQ867699-PQ867700. Additional details are available in Figs. S1–S5 and Tables S1–S8.
